# Solid-state optical refrigeration to sub-100 Kelvin regime

**DOI:** 10.1038/srep20380

**Published:** 2016-02-05

**Authors:** Seth D. Melgaard, Alexander R. Albrecht, Markus P. Hehlen, Mansoor Sheik-Bahae

**Affiliations:** 1 Dept. of Physics and Astronomy, University of New Mexico, Albuquerque, NM 87131, USA; 2 Air Force Research Laboratories, Albuquerque, NM, USA; 3 Los Alamos National Laboratory, Los Alamos, NM 87545, USA

## Abstract

Since the first demonstration of net cooling twenty years ago, optical refrigeration of solids has progressed to outperform all other solid-state cooling processes. It has become the first and only solid-state refrigerator capable of reaching cryogenic temperatures, and now the first solid-state cooling below 100 K. Such substantial progress required a multi-disciplinary approach of pump laser absorption enhancement, material characterization and purification, and thermal management. Here we present the culmination of two decades of progress, the record cooling to ≈ 91 K from room temperature.

Laser cooling in solids is accomplished through the exchange of photons via anti-Stokes fluorescence^1^,^2^,^3^,^4^,^5^,^6^,^7^,^8^. The cooling cycle proceeds as follows: low entropy photons, provided by a narrow-linewidth source, such as a laser, tuned to energies slightly less than the mean fluorescence energy, are absorbed by an atomic transition that is coupled to the vibrational modes of the lattice. The excitation leaves the system in a non-equilibrium state. Thermal equilibrium is then established through the absorption of lattice phonons, followed by blue-shifted radiative decay, or fluorescence. The resulting broadband and isotropic fluorescence, once it escapes the solid, carries higher entropy as well as higher energy compared to the excitation source and consequently leads to thermal bath cooling of the system. Realization of net cooling and subsequent refrigeration to low temperatures necessitates materials with a very high quantum (fluorescence) efficiency as well as extremely high purity so that heating from non-radiative decay and parasitic absorption do not overwhelm the cooling from the anti-Stokes fluorescence. These requirements manifest in the cooling efficiency 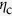 derived as the ratio of the cooling power, 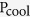, to the absorbed power, 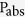
7:


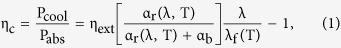


where λ is the laser wavelength, and 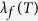 is the mean fluorescence wavelength. The external quantum efficiency 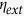 describes the fraction of atomic excitation that leads to fluorescence photons escaping from the system. The bracketed term, also known as the absorption efficiency, describes the fraction of absorbed pump power by the resonant transition 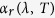 over the total absorption that includes constant parasitic (background) absorption, 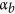.

The first net cooling 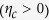 in a solid was observed in 1995^1^ in an ytterbium-doped fluorozirconate glass (ZBLANP:Yb^3+^) excited at 1030** **nm by a laser. The rare-earth ions, and in particular Yb^3+^, were suggested earlier for their extremely high quantum efficiency^9^,^10^. It was the choice of a high purity ZBLANP glass host, originally developed for long-haul optical fiber applications that ultimately made this experimental observation a reality. The laser cooling of ZBLAN:Yb glass progressed, and cooling to 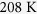 was reached in 2005^11^. However, it was not until the development of high purity Yb^3+^-doped YLiF_4_ (YLF) crystals that the first cryogenic operation was reported in 2010^2^. In addition to high purity, it was the combination of a narrow ground-state multiplet, the property of YLF to be highly doped with Yb^3+^ without quenching, and much reduced inhomogeneous broadening in the crystal that made YLF a superior host to ZBLAN. Further thermal management and enhanced absorption led to a demonstration of laser cooling to 114 K, reaching NIST’s designated range of cryogenic temperatures (<123 K) for the first time^3^. Here we report another major milestone in solid-state optical refrigeration by cooling a 10% Yb^3+^-doped YLF crystal to 91 K, the world’s first all-solid-state cryo-cooler device at sub-100 K temperatures. Optical refrigeration, as the only solid-state cryo-cooling technology, is now rapidly advancing towards applications in which vibration-free, compact, and reliable refrigeration play an essential role. Examples of such applications that benefit immediately from this technology range from reducing dark current in space-borne IR and gamma-ray sensors to cooling reference cavities for ultra-stable lasers^12^.

Rare-earth ions in low index, transparent hosts that have low vibrational energies (such as fluorides) are ideally suited to realize high external quantum efficiencies. Improvements in cooling efficiency in such materials have been successfully guided by lowering the parasitic background absorption, 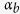. It was recently determined that the dominant source of parasitic absorption at the wavelengths of interest in YLF:Yb crystals was iron (Fe^2+^) contamination^4^. It was further deduced that such contamination may potentially be introduced by the YF_3_ starting material^13^. The findings have played a key role in further purification efforts of the starting materials for YLF crystal growth. We recently acquired a YLF:10% Yb^3+^ crystal grown by the Czochralski method^14^,^15^ with a record-high purity measured at 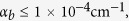 which represents a two-fold improvement from our previous best material^3^.

Full characterization of the crystal cooling performance requires measurements of the four quantities 
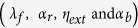
 of the cooling efficiency in equation (1), including both temperature and wavelength dependencies. The sample is placed inside a closed-cycle helium cryostat, where un-polarized temperature-dependent spectra, as well as polarized spectra at both 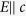 and 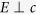 are collected between 300 K and 30 K. The mean fluorescence wavelength 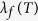 is calculated by taking the first moment of the un-polarized fluorescence spectra. Polarized absorption spectra a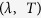 are then obtained by exploiting the concept of reciprocity via the respective polarized emission spectra 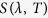 through the McCumber relation^16^,


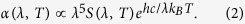


The two remaining quantities, namely the external quantum efficiency, *η*_*ext*_, and the parasitic background absorption, *α*_*b*_, are assumed to be temperature independent, and therefore are measured only at room temperature using a laser calorimetric method referred to as laser-induced temperature modulation spectrum (LITMoS), which measures the relative temperature changes induced by a tunable laser source. Temperature changes, measured by an uncooled bolometric thermal camera, are proportional to the cooling efficiency in equation (1) when normalized by absorbed power. Here, a continuous wave Ti:Sapphire laser with a tuning range from 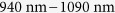 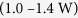 induces heating at wavelengths shorter than the mean fluorescence wavelength and cooling at longer wavelengths. The quantities for the present YLF:10% Yb^3+^ crystal measured using the LITMoS method are 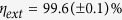 and 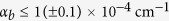. It is important to note that when the background absorption values are reduced to <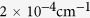, the induced heating at long wavelengths is very small, requiring increased pump laser absorption to generate a measurable temperature change. Therefore, laser-induced heating measurements were taken by placing the crystal inside a non-resonant cavity that increases the total pump absorption by a factor of ≈ 20, allowing for increased signal-to-noise measurements on the thermal camera. Even with increased absorption, the heating effects from such small background absorption are difficult to measure, allowing us to only define an upper bound for 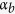.

With the four ingredients of the cooling efficiency determined, we calculate and plot the cooling efficiency 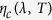 contour map as shown in Fig. 1. The boundary between light-blue and yellow corresponds to 
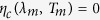
, separating the cooling (blue, 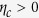) from the heating (red, 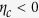) regimes. The valley of the blue region at a temperature 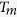 denotes the minimum achievable temperature (MAT) at pump wavelength 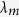. From the cooling map in Fig. 1, we find that the lowest MAT (or global MAT) is predicted to be ≈ 89 K occurring at 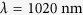. This corresponds to the 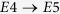 transition of the Yb^3+^ ion in the crystalline YLF host (see inset in Fig. 1)^2^.

In order to approach the MAT for a given crystal, it is necessary to minimize all external heat loads, and to maximize the pump laser absorption. Heat load mitigation requires minimizing the convective, conductive, and radiative heat loads. Both the convective and conductive heat loads are minimized by placing the sample inside a vacuum chamber evacuated to 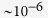
torr and supporting it on the tips of six thin 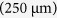 fibers that are each filed to a point. The radiative heat load is the dominant load and requires special attention. For a sample inside a chamber (subscript s and c, respectively) with emissivity 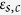 and surface area 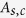 at temperature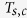, the radiative heat load 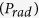 is given by,


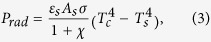


where 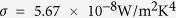 is the Stefan-Boltzmann constant and


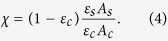


For a given sample with known surface area that is cooled inside a chamber whose walls are held at (or near) room temperature, the only remaining heat load reduction can be achieved by maximizing *χ*. This is accomplished by reducing the chamber area and emissivity. Therefore, a specially designed copper “clamshell” is machined to fit tightly around the cooling sample to reduce the chamber surface area. It is then coated with a solar-selective coating (Acktar Nano Black), which is designed to absorb the high power anti-Stokes fluorescence generated by the crystal to prevent reabsorption, and to maintain low thermal emissivity. Therefore, the coating appears black under visible light and highly reflective at thermal wavelengths.

After heat load mitigation, the next step is to maximize the pump light absorption. This is accomplished by using a 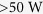 continuous wave linearly-polarized fiber laser centered at 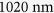 with a linewidth of ≈ 0.5 nm (custom-made, IPG Photonics Inc.). The laser, after passing through a Faraday isolator, is trapped inside a non-resonant cavity (folded optical delay line or Herriott cell^17^), constructed by a 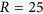 cm spherical mirror and a flat mirror. The pump laser is coupled into this cavity through a 500 μm diameter hole drilled into the flat mirror. By adjusting the launch angle of the pump beam with respect to the axis of the Herriott cell, a circular pattern of pump spots is formed whose number represents the number of trapped roundtrips. This cavity allows for 22 passes (11 spots) through a Brewster-cut crystal of dimension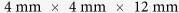, which represents an increase of ≈ 8 passes over previous efforts. This, in turn, implies an increase of the absorbed power from 27.1% to 39% at 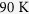
4. Note that the absorption is strongly temperature dependent: while >90% of the total incident pump power is absorbed in ≈ 4 passes at room temperature, only 39% of the pump power is absorbed after all 22 passes at 90 K. Consequently, provisions were made for the remaining pump light 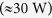 to safely exit the cavity through the original entrance hole when the crystal is cold and under vacuum. Furthermore, ensuring that all of the 22 passes traverse the crystal becomes a challenge. The alignment is performed in air (hence near room temperature) where the laser power must be reduced sufficiently to prevent cooling the crystal to the point of condensation. Therefore, a Ti:Sapphire guide laser tuned to 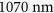, a low absorption region in YLF:Yb, co-propagates with the pump to provide a means to reliably count the number of passes through the crystal, where spots of scattered light can be seen through an IR viewer at the point of reflection on the mirror, and to ensure the safe exit of the remaining pump light once the crystal is cold. An additional verification of passes can be made when the crystal becomes cold. This is due to decreased absorption, leading to increased intensity for subsequent passes in the cooling crystal, where a small amount light leaks through the high reflectivity back mirror, transmits out of the vacuum chamber through a window, and is monitored with an IR detector card. As stated earlier, each spot on the card represents two passes through the crystal, and provides an excellent method for counting passes as well as determining if any misalignment occurred via scattered or missing spots. An example image is provided in Fig. 2 where eight spots can be seen, corresponding to 16 successful passes, whereas the best performing experiment had eleven spots.

A simplified experimental setup is shown in Fig. 2 where a YLF:Yb crystal is tightly enclosed by a heat sunk clamshell (cutaway for viewing the crystal) and placed inside a vacuum chamber. The laser enters the chamber through an AR coated window, and is trapped inside the non-resonant cavity. Multiple passes through YLF:Yb crystal can be seen, resulting in increased absorption.

Differential luminescence thermometry (DLT) is used as a sensitive, non-contact temperature measurement^18^, since thermal cameras are ineffective below 250 K, and directly connected devices will alter the temperature measurement. In this measurement, temperature-dependent spectra are obtained in real time, normalized, and referenced to a corresponding spectrum acquired at room temperature. The differential signal is defined as


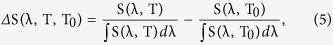


where normalization to an integrated area of each spectrum eliminates input power fluctuations. Special attention must be paid to avoid the spectral area (if any) affected by laser scatter. The scalar DLT signal is the absolute area of the differential spectrum,


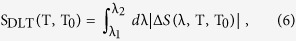


where the limits of integration bracket the spectral emission of the YLF:Yb^3+^ crystal. This signal is then converted into an absolute temperature through a separate calibration process where temperature dependent emission spectra are collected from 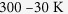
(mentioned earlier). Values of 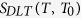 are calculated from the known temperatures and used to create a calibration curve.

At 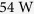 of incident pump power, cooling to 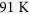 was achieved (blue curve in Fig. 3) with an estimated heat lift of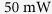, settling into thermal equilibrium with the surrounding heat load on the crystal. Variations in the clamshell temperature (Fig. 3 red curve) are the result of temperature-dependent absorption in the crystal. Initially, high-power fluorescence generated by complete pump absorption is deposited into the clamshell at time 0, accounting for the sudden increase in temperature. As the crystal cools, the temperature-dependent absorption inside the crystal reduces, lowering the fluorescence power and leading to a slowly reduced clamshell temperature. The initial clamshell temperature was adjusted below room temperature to help further reduce the external heat load in order to achieve temperatures closer to the predicted MAT. Maintaining the clamshell temperature at 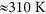 resulted in cooling to 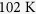 with an estimated heat lift of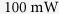, corresponding well with the relative increase in radiative heat load. This demonstrates the need for increased pump absorption in order to demonstrate temperatures near MAT without manually lowering the clamshell temperature to reduce external heat load. Estimations of cooling power are determined from Eq. 1, where absorbed power and cooling efficiency have been previously determined.

In conclusion, substantial progress has been made in optical refrigeration since net cooling was first demonstrated 20 years ago. Here we present the culmination of progress, the first double digit solid-state refrigeration by cooling a YLF:10% Yb^3+^ crystal by anti-Stokes fluorescence to 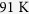 with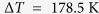. To achieve these record results, a YLF:Yb crystal having the lowest measured background absorption to date was grown. It was placed inside an improved non-resonant cavity that enhanced pump light absorption, accompanied by extensive efforts to minimize external heat loads. These results push optical refrigeration into the regime where a practical application is the next step.

Device cooling applications will require overall enhancement of efficiency. Since it was determined in this work that enhanced pump light is necessary to achieve lower temperatures, the next most promising step is to further enhance laser trapping by exploiting an astigmatic Herriott cell. This will provide an estimated increase of incident pump light absorption from 39% to >90% at low temperatures, both removing the need for manual temperature reduction of the clamshell and increasing the heat load capacity for an all solid-state optical cryo-cooler. Additional efficiency gains will be found in the purification of host materials and using other rare-earth ions and hosts.

## Additional Information

**How to cite this article**: Melgaard, S. D. *et al*. Solid-state optical refrigeration to sub-100 Kelvin regime. *Sci. Rep.* **6**, 20380; doi: 10.1038/srep20380 (2016).

## Figures and Tables

**Figure 1:**
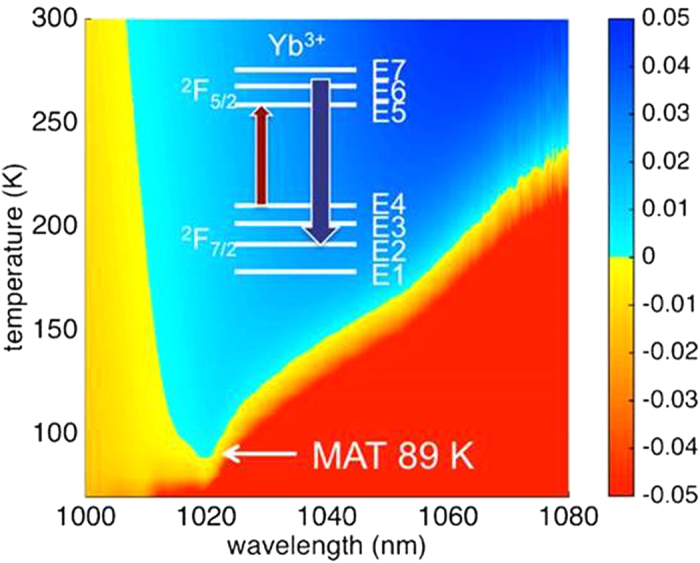
Cooling efficiency contour map 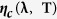 evaluated for the latest high purity YLF:10% Yb^3+^crystal. Blue regions denote cooling, and red regions denote heating. The minimum achievable temperature (MAT) of 89 K is highlighted. (Inset) Illustration of the Yb^3+^ ion energy level diagram (not to scale) with example absorption (red) and anti-Stokes emission (blue) arrows.

**Figure 2:**
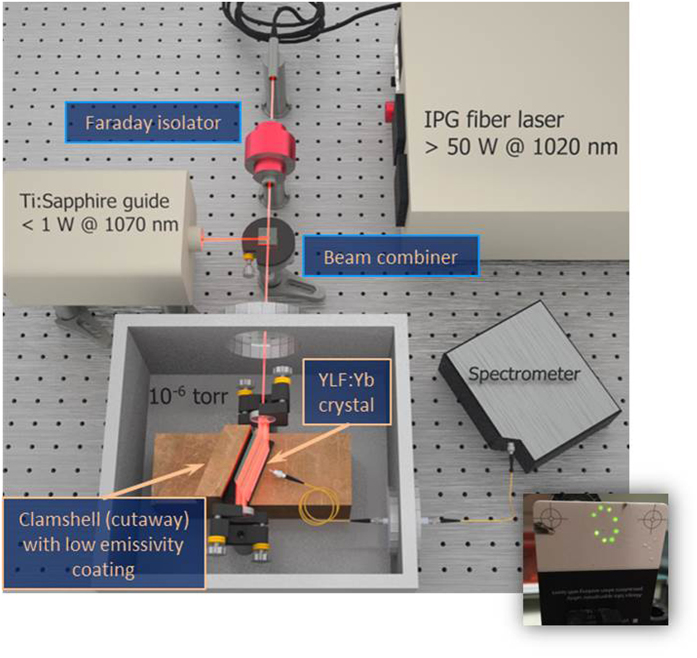
Simplified experimental setup. The IPG pump laser providing >50 W at 1020 nm is isolated and combined with a Ti:Sapphire guide laser tuned to 1070 nm. Both lasers propagate collinearly into a non-resonant (NR) cavity where the YLF:Yb cooling crystal resides inside a clamshell (cutaway for viewing the crystal) in a vacuum chamber held at 10^−6^ torr. A fiber collects fluorescence into a spectrometer where temperature is deduced through DLT. (inset) An image of an IR card where eight laser spots can be seen, corresponding to 16 successful passes, whereas the best performing experiment had 22 passes.

**Figure 3:**
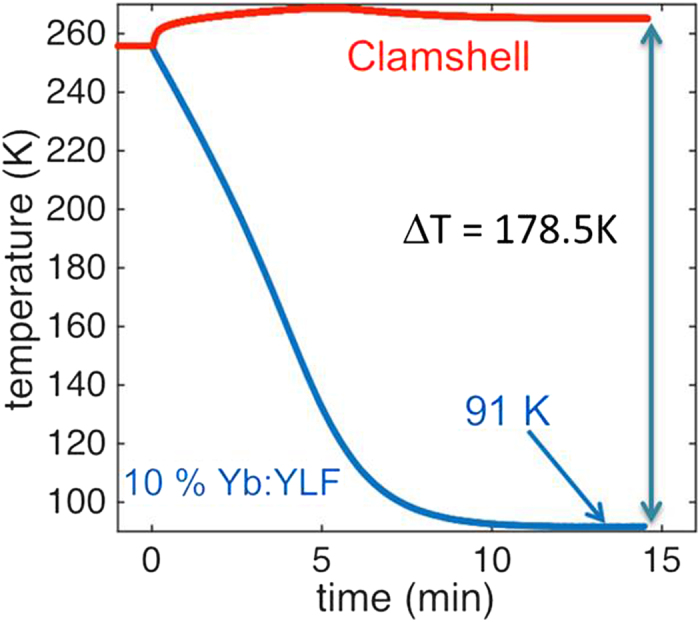
Record cooling result. The crystal temperature (blue) reaches 91 K after ≈ 12 minutes of pumping while the clamshell temperature is maintained at ≈ 265 K.
